# Comparison of Hi-C results using in-solution versus in-nucleus ligation

**DOI:** 10.1186/s13059-015-0753-7

**Published:** 2015-08-26

**Authors:** Takashi Nagano, Csilla Várnai, Stefan Schoenfelder, Biola-Maria Javierre, Steven W. Wingett, Peter Fraser

**Affiliations:** Nuclear Dynamics Programme, The Babraham Institute, Cambridge, CB22 3AT UK

## Abstract

**Background:**

Chromosome conformation capture and various derivative methods such as 4C, 5C and Hi-C have emerged as standard tools to analyze the three-dimensional organization of the genome in the nucleus. These methods employ ligation of diluted cross-linked chromatin complexes, intended to favor proximity-dependent, intra-complex ligation. During development of single-cell Hi-C, we devised an alternative Hi-C protocol with ligation in preserved nuclei rather than in solution. Here we directly compare Hi-C methods employing in-nucleus ligation with the standard in-solution ligation.

**Results:**

We show in-nucleus ligation results in consistently lower levels of inter-chromosomal contacts. Through chromatin mixing experiments we show that a significantly large fraction of inter-chromosomal contacts are the result of spurious ligation events formed during in-solution ligation. In-nucleus ligation significantly reduces this source of experimental noise, and results in improved reproducibility between replicates. We also find that in-nucleus ligation eliminates restriction fragment length bias found with in-solution ligation. These improvements result in greater reproducibility of long-range intra-chromosomal and inter-chromosomal contacts, as well as enhanced detection of structural features such as topologically associated domain boundaries.

**Conclusions:**

We conclude that in-nucleus ligation captures chromatin interactions more consistently over a wider range of distances, and significantly reduces both experimental noise and bias. In-nucleus ligation creates higher quality Hi-C libraries while simplifying the experimental procedure. We suggest that the entire range of 3C applications are likely to show similar benefits from in-nucleus ligation.

**Electronic supplementary material:**

The online version of this article (doi:10.1186/s13059-015-0753-7) contains supplementary material, which is available to authorized users.

## Background

Chromosome conformation capture (3C) and its various derivatives such as 4C (circularized chromosome conformation capture), 5C (carbon-copy chromosome conformation capture) and Hi-C have emerged as standard tools to analyze the three-dimensional organization of the genome in the nucleus [1, 2]. These methods have been extensively used for addressing various biological questions and subject to further technical developments [3–8], contributing substantially to our understanding of nuclear genome organization. All these methods depend on a simple principle of proximity-dependent ligation where DNA ends in cross-linked, restriction-digested, solubilized chromatin complexes are subjected to re-ligation in dilute solution intended to favor stochastic re-ligation of nearby DNA fragment ends in the same complex [9]. DNA fragment pairs that are ligated are the direct readout and form the basis for 3C-based assay results. Frequent ligation between two fragments is interpreted to indicate that those fragments were in spatial proximity in vivo in a significant proportion of cells at the time of cross-linking, thus providing clues to the three-dimensional organization of the genome. Therefore, proximity-dependent ligation is a critical step in 3C and all of its derivative methods such as 4C, 5C and Hi-C.

It has been regarded as essential to extensively dilute the solubilized cross-linked chromatin prior to ligation to prevent non-specific ligation due to chance inter-molecular collisions. However, during our recent development of single-cell Hi-C [10] we modified the original Hi-C procedure and carried out the ligation step within preserved nuclei. Although the chromatin is not physically diluted when the ligation takes place in this modified procedure, we confirmed a high correlation between the results of the original “in-solution ligation” and our modified “in-nucleus ligation” procedures [10]. This is consistent with Comet et al. [11], who found that dilution prior to ligation is not essential to observe characteristic 3C profiles, and Gavrilov et al. [12], who found that most of the chromatin remains insoluble in diluted 3C samples and that the bulk of the 3C signals come from chromatin ligation in this insoluble fraction.

We observed that Hi-C coverage appeared to be more uniform in single-cell Hi-C, suggesting that in-nucleus ligation may actually improve Hi-C results [10]. In-nucleus ligation Hi-C has also been employed by Sofueva et al. [13] and Rao et al. [14]. In particular, Rao et al. reported interaction maps at higher resolution after deep sequencing than previous in-solution ligation methods, further suggesting that in-nucleus ligation may lead to improved results. Here we extensively compare the two ligation methods side by side, and find that in-nucleus ligation provides more consistent ligation frequency over the full range of genomic distances, and produces data with significantly less bias and significantly less technical noise.

## Results

An overview of the Hi-C library method employing either in-solution ligation or in-nucleus ligation is shown in Additional file 1. In-solution Hi-C ligation is essentially as described by Lieberman-Aiden et al. [15] and includes a sodium dodecyl sulfate (SDS) treatment to lyse nuclei and solubilize chromatin complexes prior to ligation. The in-nucleus ligation procedure omits the SDS/lysis step and executes ligation in preserved nuclei.

To compare the two methods, we split cell pellets into different aliquots and carried out the Hi-C procedure in parallel; fixation, restriction digestion and biotin fill-in, up until the ligation step. In half the samples we lysed the cells for in-solution ligation, and with the other half we performed in-nucleus ligation. Downstream Hi-C library preparation steps after the ligation were identical for both aliquots of cells. We created Hi-C libraries in this way from two biological replicates each of mouse foetal liver cells (mouse-1 and mouse-2) and human embryonic stem (ES) cells (human-1 and human-2). We also sequenced a random ligation library prepared by reversal of the cross-links and purification of the DNA prior to ligation.

### In-nucleus ligation reduces noise

**Fig. 1:**
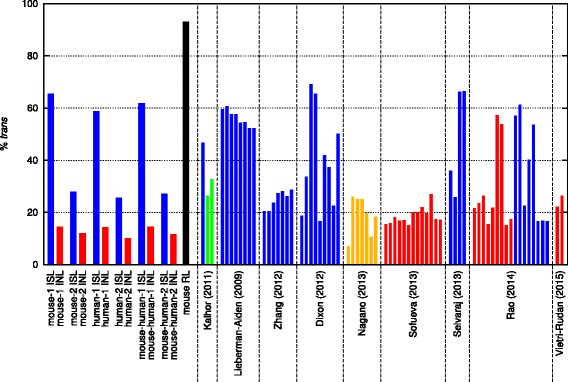
The frequencies of mapped and filtered *trans*-chromosomal di-tags (*%trans*). The percentage of *trans*-chromosomal di-tags in the Hi-C datasets in this study employing in-solution ligation (*ISL*; *blue*), in-nucleus ligation (*INL*; *red*) and random ligation (*RL*; *black*). Additional datasets from the publications indicated are shown with *blue* (in-solution ligation), *red* (in-nucleus ligation), *green* (TCC) or *orange* (single-cell Hi-C with in-nucleus ligation)

After paired-end sequencing of the Hi-C libraries, the resulting FASTQ files were mapped against either the mm9 or hg19 genome assemblies using HiCUP [16], a Hi-C bioinformatics pipeline for aligning Hi-C reads and removing commonly encountered experimental artefacts. Table 1 shows the numbers of total di-tags and mapped di-tags for each library along with the breakdown of unique di-tags including the percentages of intra-chromosomal (*cis*) and inter-chromosomal (*trans*) di-tags for each dataset. The most obvious initial observation is that the in-nucleus datasets have consistently, markedly lower rates of *trans*-chromosomal interactions (10–14 %) compared with their corresponding in-solution ligation datasets (26–65 %). The relatively high percentage of *trans*-chromosomal contacts from in-solution ligation is consistent with several previously published in-solution ligation Hi-C datasets [3, 4, 10, 13–15, 17–20] (Fig. 1). As expected, the random ligation dataset had greater than 90 % *trans*-chromosomal contacts (Table 1 and Fig. 1). We also compared published data from tethered conformation capture (TCC) [4] and found the percentage of *trans*-chromosomal contacts to be intermediate between in-solution and in-nucleus Hi-C ligation [10, 13, 14, 20]. We hypothesized that the lower percentage of *trans*-chromosomal ligations from the in-nucleus ligation libraries may represent a reduction in technical noise, and that the in-solution ligation conditions may create the possibility for increased random ligation events, which would appear primarily as *trans*-chromosomal contacts. Such random ligation events would be expected to contribute to technical noise that may obscure some of the finer features in the Hi-C datasets.

**Table 1:** Datasets in this study

Dataset name	Total di-tags	Mapped di-tags	Percent_re-ligation_	Unique di-tags	Percent_*cis*_	Percent_*trans*_	Percent_*hybrid*_
Mouse-1 ISL	255,402,369	173,621,670	2.17	123,691,456	34.7	65.3	0.02
Mouse-2 ISL	131,079,448	83,009,025	3.85	32,939,021	72.1	27.9	0.05
Human-1 ISL	31,550,241	19,444,276	5.61	11,128,672	41.3	58.7	0.08
Human-2 ISL	27,518,246	17,060,401	7.79	6,994,931	74.5	25.5	0.03
Mouse-human-1 ISL	40,398,555	22,667,233	3.16	17,137,291	38.2	61.8	11.9
Mouse-human-2 ISL	2,928,112	1,405,001	3.84	488,340	72.9	27.1	2.9
Mouse-1 INL	242,699,915	167,175,980	3.49	108,733,338	85.6	14.4	0.16
Mouse-2 INL	134,813,901	85,804,344	6.80	37,259,526	88.0	12.0	0.02
Human-1 INL	34,860,295	22,113,388	10.40	11,457,531	85.8	14.2	0.07
Human-2 INL	33,500,802	20,280,022	14.03	7,777,001	90.0	10.0	0.03
Mouse-human-1 INL	33,762,441	19,318,810	4.16	11,533,994	85.6	14.4	0.06
Mouse-human-2 INL	2,920,332	1,423,890	7.53	573,366	88.5	11.5	0.07
Mouse RL	36,268,145	25,581,886	9.25	4,920,447	7.1	92.9	-

*INL* in-nucleus ligation, *ISL* in-solution ligation, *RL* random ligation

**Fig. 2:**
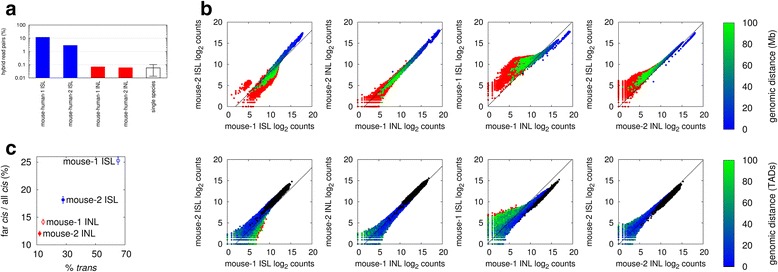
In-nucleus ligation reduces noise from Hi-C datasets. **a** The frequencies of hybrid mouse-human di-tags obtained from the mixture of mouse and human cells by in-solution (*ISL*; *blue*) and in-nucleus (*INL*; *red*) ligation experiments, compared with the mean hybrid di-tag frequencies in unmixed mouse or human samples (single species; *white*, with standard deviation). **b** Scatter plots comparing the log_2_ binned interaction counts for mouse datasets at 10 Mb resolution (*top panels*), and topologically associated domain (TAD) scale (*bottom panels*). Colors represent interaction distances according to the color bar shown; *red dots* represent *trans*-chromosomal interactions, *black dots* represent intra-TAD interactions in *bottom panels. Dashed lines* show the interaction counts corrected for the difference in the total counts. **c** The ratio of far-*cis* (>20 Mb) to all *cis*-chromosomal interaction counts plotted against the ratio of *trans*-chromosomal to all interaction counts (Pearson R > 0.98)

Ideally, Hi-C ligations should only occur between fragments within a single, cross-linked, chromatin complex. Any ligation products formed between different cross-linked complexes are likely to be between fragments that were not in proximity and would therefore contribute to noise in the data. In fact, if ligation occurs between fragments in different complexes, it is most likely that those complexes come from different cells. To test the hypothesis that in-nucleus ligation creates less noise in Hi-C data compared with in-solution ligation, we created Hi-C libraries from pools of cells composed of a 5:1 mixture of mouse:human cells by either in-solution or in-nucleus ligation. We then compared the frequencies of hybrid di-tags, where mouse and human genomic sequences were ligated together. We found remarkably high levels of hybrid di-tags in the two in-solution ligation datasets; 2.9 % and 11.9 % (Table 1 and Fig. 2a). In sharp contrast we found 30–100-fold lower hybrid di-tags (<0.1 %) in the in-nucleus ligation replicates. The mapping results derived from non-mixed libraries containing cells of a single species show similarly low percentages of hybrid di-tags (average 0.06 %; Fig. 2a), suggesting that mis-mapping could account for the majority of the hybrid di-tags observed in the in-nucleus ligation datasets, suggesting very low random ligation. For comparison, in a theoretical random ligation experiment using the same mixture of mouse and human cells, we would expect to observe approximately 28 % human–mouse hybrid di-tags (see “Materials and methods”), suggesting that in-solution Hi-C ligation produces a significant amount of random ligation between cross-linked complexes. In addition to this abundance of directly measurable human–mouse di-tags generated by in-solution ligation Hi-C, a substantial number of un-measurable spurious di-tags from different cells of the same species would be expected. In the mixing experiments we found no bias for hybrid di-tags between A and B compartments (defined by Lieberman-Aiden et al. [15]), indicating that this source of Hi-C noise is unbiased and random (Additional file 2). Collectively, these results indicate that a significant percentage (perhaps as much as 50 %) of the ligations in Hi-C datasets employing in-solution ligation could come from random or spurious ligation events. In contrast, in-nucleus ligation effectively removes this highly significant source of noise.

If the increased *trans*-chromosomal interactions generated during in-solution ligation Hi-C are caused by random ligations, far *cis*-chromosomal interactions should also contain a significant amount of noise. To test if this is the case, we compared the binned interaction counts, and topologically associated domain (TAD; see below) level interaction counts, in the raw Hi-C data between the mouse in-solution and in-nucleus ligation datasets. We used these datasets because they had the highest coverage. With increasing genomic distance we found a significant increase in the *cis*-chromosomal interaction log counts for the in-solution ligation datasets compared with the in-nucleus datasets in both the binned and TAD level analyses (Fig. 2b). This diverged from what would be expected purely from the ratio of the total number of interactions of the two datasets. We also observed this trend in comparisons of the human datasets (data not shown). Further inspection revealed a strong correlation between the ratios of *trans*-chromosomal to all interactions, and the ratios of far *cis*- (>10 Mb) and all *cis*-chromosomal interactions (Pearson R > 0.98 for the mean; Fig. 2c). Thus, in-nucleus ligation produces significantly fewer long-range *cis*-chromosomal di-tags (>10 Mb) compared with in-solution ligation, which is consistent with the hypothesis of reduced random ligation noise during in-nucleus ligation.

### In-nucleus ligation improves reproducibility

**Fig. 3:**
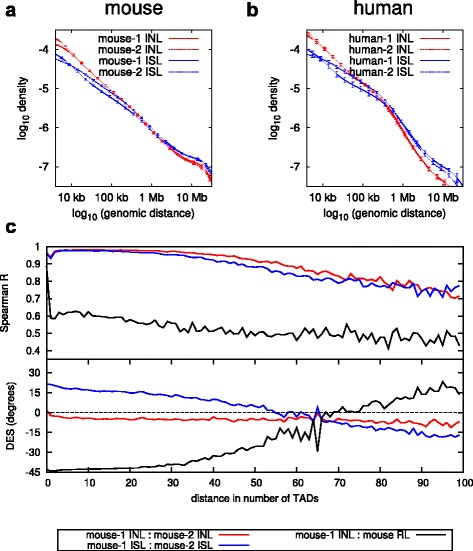
Reproducibility of *cis*-chromosomal interactions between replicates. *Cis*-chromosomal interaction frequency density as a function of the genomic distance for in-solution ligation (*ISL*; *blue*) and in-nucleus ligation (*INL*; *red*), for mouse foetal liver (**a**) and human ES cell samples (**b**). The *error bars* show one standard deviation from the mean of all chromosomes. **c** *Top panel*: Spearman correlation coefficient between replicates as a function of genomic distance. *Bottom panel*: deviation from expected slope (*DES*) as a function of genomic distance

The results shown in Fig. 2b suggest that in-nucleus ligation produces data that are more reproducible between replicates. To examine this in greater detail we compared the distribution of all *cis*-chromosomal interactions, often referred to as the powerlaw curves. We observed that the curves for in-nucleus ligation-derived datasets are significantly more reproducible between replicates compared with those of in-solution ligation-derived datasets (Fig. 3). Notably, the in-nucleus replicates are highly reproducible across the entire range, from 10 kb to 100 Mb distance, whereas the in-solution replicates show significant divergence over broad ranges. As expected from our analyses shown in Fig. 2, the in-nucleus ligation generates significantly fewer *cis*-chromosomal di-tags over long distances (>10 Mb) compared with in-solution ligation, and also showed an increase in close *cis* di-tags. In fact, the powerlaw curves corresponding to in-nucleus ligation maintain a more uniform slope over the entire range of distances.

We also assessed reproducibility between replicates by calculating the Spearman correlation coefficient (R), and the deviation from the expected slope (DES) (see “Materials and methods”). In a perfectly reproducible experiment we would expect the Spearman correlation coefficient to be R = 1 and the deviation from the expected slope to be DES = 0. As can be seen in Fig. 3c the Spearman coefficient by itself can be misleading since it suggests that the random ligation dataset correlates well with the other Hi-C datasets. However, considering the Spearman correlation together with the DES provides a better comparison. We note that both the in-nucleus and in-solution datasets have remarkably good Spearman correlation coefficients, suggesting high reproducibility at multiple length scales. However, the DES calculation shows that the in-solution ligation comparison deviates substantially from the expected slope, whereas the in-nucleus ligation comparison deviates only marginally, indicating improved reproducibility. Thus, in combination with the results shown in Fig. 2 we conclude that in-nucleus ligation provides more consistent results with more uniform coverage of *cis*-chromosomal interactions compared with in-solution Hi-C.

### Reduced fragment length bias

**Fig. 4:**
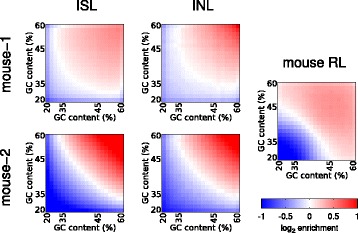
Experimental GC content bias. The mouse in-solution (*ISL*), in-nucleus (*INL*) and random (*RL*) ligations are compared for GC content-related bias matrices, calculated using the Hi-C matrix correction [21], employing a 100-kb bin resolution

**Fig. 5:**
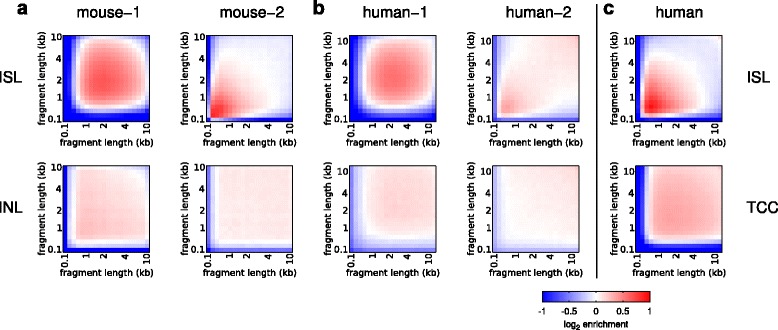
Experimental fragment length bias. The mouse and human in-solution (*ISL*), in-nucleus (*INL*) and TCC ligation datasets are compared for fragment length bias matrices, calculated using the Hi-C matrix correction [21], employing a 100-kb bin resolution. **a** Mouse foetal liver. **b** Human ES cells. **c** GM12878 human lymphoblastoid cells [4]

Hi-C experiments are subject to systematic experimental biases, such as restriction fragment length and the GC content biases [21] that can affect the coverage of particular restriction fragments. These biases can be corrected by normalization of the Hi-C matrix containing the binned interaction data by inferring the bias contributions explicitly [21] or by a matrix balancing algorithm [22]. We found above that in-nucleus ligation produces more consistent *cis*-chromosomal interactions than in-solution ligation when considering unnormalized data. We tested if in-nucleus ligation improves the systematic biases discovered by Yaffe and Tanay [21]. We found little difference in the GC bias comparing in-solution and in-nucleus ligation (Fig. 4). Since the GC content bias is mainly created during library amplification PCR [23], this bias would be expected using both methods. However, we found that in-nucleus ligation results in a remarkable reduction in fragment length bias (Fig. 5a, b), generating more uniform ligation between fragments regardless of length with the exception of extremely short (<100 bp) fragments. TCC appears more uniform compared with in-solution ligation, but it still exhibits a strong bias between short and long fragment lengths (Fig. 5c). We conclude that in-nucleus ligation effectively removes restriction fragment length bias, producing more consistent results between replicates.

### Improved reproducibility of normalized Hi-C data

**Fig. 6:**
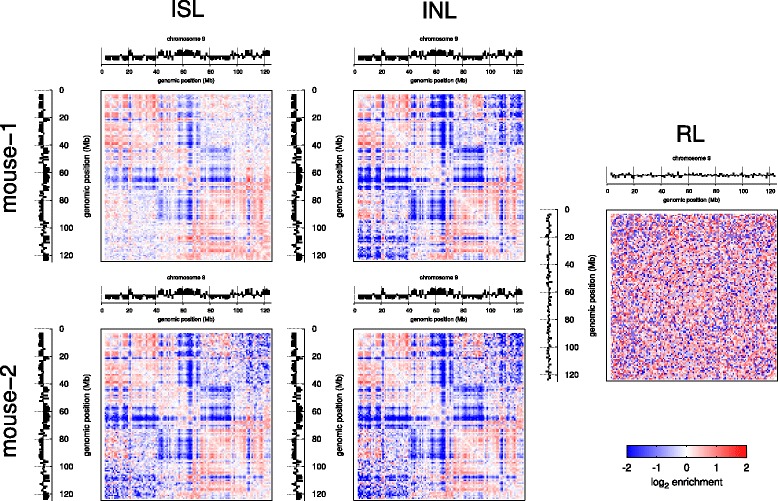
Normalized Hi-C matrices with compartments. Normalized matrices for mouse chromosome 9 from the indicated datasets with the first principal component indicated A and B compartments (defined by Lieberman-Aiden et al. [15]), at the *top* and *left* of each map. *INL* in-nucleus ligation, *ISL* in-solution ligation, *RL* random ligation

**Fig. 7:**
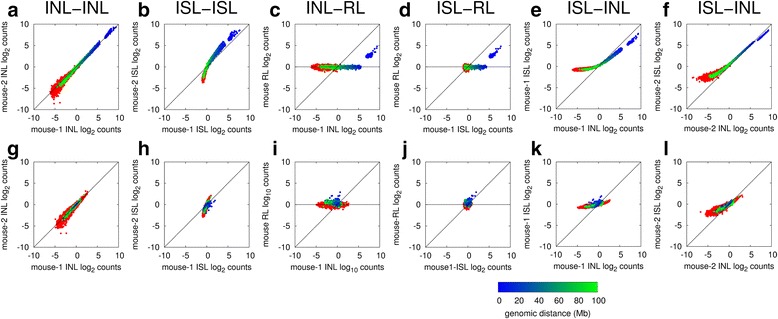
Reproducibility of the corrected Hi-C matrices. Element-wise comparison of coverage-corrected (**a**–**f**) and coverage- and distance-corrected (**g**–**l**) Hi-C matrices as indicated. The scatter plots show the log_2_-corrected counts in one dataset against the corresponding count values in the second dataset, for all *cis*-chromosomal (*blue* to *green* color varying with genomic distance) and *trans*-chromosomal (*red*) bin interaction counts. The correction of Imakaev et al. [22] was applied, using a bin resolution of 10 Mb. *INL* in-nucleus ligation, *ISL* in-solution ligation, *RL* random ligation

To investigate the reproducibility in close *cis*-, far *cis*- and *trans*-chromosomal di-tags in the normalized data, we plotted the normalized Hi-C matrices for chromosome 9 with the first principle component indicating the A and B compartments defined by Lieberman-Aiden et al. [15] (Fig. 6). We found that the matrices were similar and the compartments were identical at 1-Mb resolution; however, we noted that the in-nucleus ligation matrices appeared sharper, which is particularly obvious at long range. To investigate this further, we plotted the coverage-corrected (Fig. 7a–f), and coverage- and distance-corrected (Fig. 7g–l) Hi-C matrix element values from the different datasets against each other, as in Fig. 2. All show high reproducibility for close *cis*-chromosomal di-tags (blue dots) whereas far *cis*- and *trans*-chromosomal di-tags (green and red dots) show high reproducibility only with in-nucleus ligation (Fig. 7a, g). In contrast, the in-solution ligation datasets display poor reproducibility for far *cis*- and *trans*-chromosomal di-tags (Fig. 7b, h). We observed similar effects when comparing the raw interaction counts (Fig. 2b), although it is more pronounced after correction for technical bias.

Comparison of the Hi-C data with random ligation data shows a clear shift in the scatter plot toward the horizontal axis, away from the marked diagonal (Fig. 7c, d, i, j). This trend is due to the increased noise in the random ligation. When comparing in-solution with in-nucleus datasets we also observed a similar shift away from the diagonal, suggesting that the in-solution ligation datasets have increased noise (Fig. 7e, f, k, l). In fact, this shift away from the diagonal is already apparent when comparing the two in-solution datasets (Fig. 7b, h). Together with the observations shown in Figs. 1, 2 and 3, these results suggest that in-nucleus ligation results in reduced noise and increased reproducibility of the normalized data.

### Sharper structural features

**Fig. 8:**
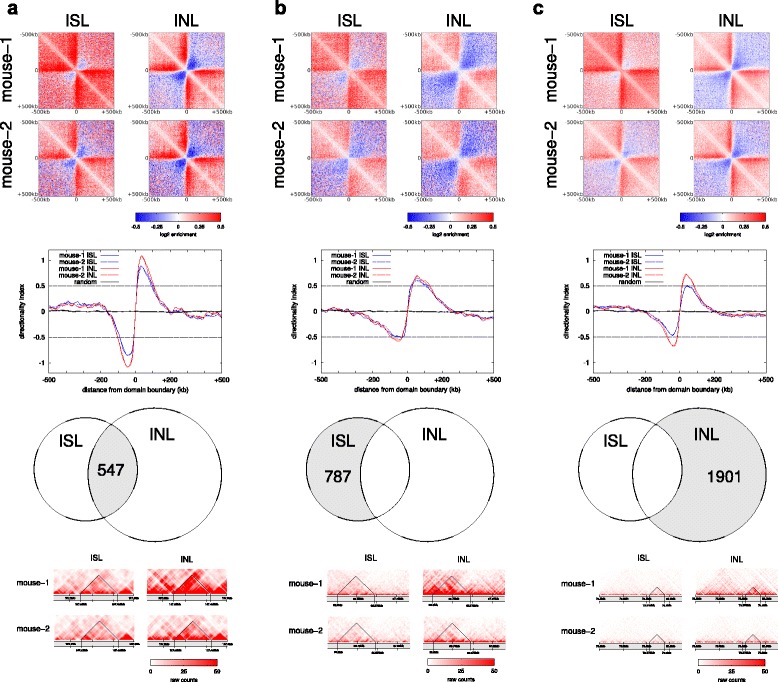
Comparison of TAD boundary recognition. Average coverage- and distance-corrected Hi-C interaction profiles around TAD boundaries (*top panels*). Averaged standard score of the interaction directionality indices around TAD boundaries (*line graphs*). *Venn diagrams* of boundaries detected in the datasets as shown. Zoomed in views of randomly selected TADs from mouse chromosome 9 for each category (*bottom panels*). **a** TAD boundaries detected in both in-nucleus ligation (*INL*) and both in-solution ligation (*ISL*) replicates. **b** TAD boundaries detected by both ISL replicates only. **c** TAD boundaries detected by both INL replicates only

Hi-C results provide information on chromatin organization into TADs [18, 24, 25]. TAD boundaries are defined by the observation that *cis*-chromosomal interactions within each TAD are more abundant over those connecting adjacent TADs. Our finding that in-nucleus ligation improves Hi-C data quality by reducing noise and bias prompted us to test if in-nucleus ligation datasets define these structural features more clearly. We identified TAD boundaries by calculating the directionality index of Hi-C interactions in the two in-nucleus mouse foetal liver replicates and their corresponding in-solution datasets. We found that approximately 60 % of the TAD boundaries previously identified by Dixon et al. [18] in mouse ES cells were within 100 kb of the boundaries we identified with the in-solution ligation datasets, whereas 70 % were within 100 kb of our identified in-nucleus ligation boundaries. We identified 2448 TAD boundaries that were consistent between the two in-nucleus datasets. In contrast, we found 1334 TAD boundaries that were consistent in both in-solution datasets. We selected 547 TAD boundaries that were defined by all four datasets and analyzed the distribution of di-tags and interaction directionality indices around these boundaries (Fig. 8a). The in-nucleus ligation results consistently show stronger depletion of interactions across the selected boundaries, demonstrating a more robust recognition of these structural features. The boundaries found only by in-solution ligation were weak boundaries, very close to the detection threshold (Fig. 8b). Although these boundaries were not detected by both in-nucleus replicates, on average they appear equally apparent with in-nucleus ligation. As expected, the boundaries found only by in-nucleus ligation Hi-C did not, on average, reach the detection threshold with in-solution ligation (Fig. 8c). These results are in agreement with the greater number of boundaries identified consistently by in-nucleus ligation (2448) compared with in-solution ligation (1334). We conclude that in-nucleus ligation is capable of highlighting domain boundaries and potentially other structural features more reproducibly.

## Discussion

We have shown that in-nucleus ligation results in significantly improved Hi-C data in many aspects. We found a significant reduction in technical noise arising from spurious ligation events, and increased reproducibility between replicates for *trans*-chromosomal and *cis*-chromosomal interactions at all distances. In addition, in-nucleus ligation effectively eliminates the fragment length bias normally found in conventional Hi-C data. All of these improvements appear to lead to cleaner data, allowing for a more robust detection of TAD boundaries, as an example.

We think many of these aspects are related to one another. For example, the reduced technical noise coming from spurious ligations could be because the ligatable fragment ends are physically more constrained within the preserved nuclei, and thus only fragment ends in close nuclear proximity are capable of ligation. We have shown that a large amount of measurable, hybrid di-tags appear in mixing experiments using in-solution ligation, demonstrating that conventional in-solution ligation in a single sample will result in a significant amount of un-measurable spurious di-tags in *cis* and *trans*. Since these artefactual events will much more likely occur between different chromosomes, distal regions from the same chromosome or indeed between complexes from different cells, eliminating these spurious ligation events results in less noise and improved reproducibility for *trans*-chromosomal and far *cis*-chromosomal interactions. Since spurious ligations are un-measurable in a single sample, we suggest that an indicator of Hi-C library quality is a reduced percentage of *trans* di-tags.

We suggest that the results point to the benefits of a nuclear microenvironment during the ligation process. Fragment ends undergoing in-nucleus ligation may have reduced mobility compared with in-solution ligation due to molecular crowding in the nucleus. Their potential movements during the ligation process would be more restricted within a confined space, thereby increasing the chances of their ligation to spatially proximal fragment ends (Additional file 3a). In this scenario, fragments would not necessarily need to be directly cross-linked to each other to be ligated, but they simply need to be in spatial nuclear proximity. Anything that relaxes the spatial constraints between fragment ends will potentially compromise proximity-dependent ligation and result in greater noise (Additional file 3b). For example, the harsh solubilization process (65 °C in the presence of SDS) prior to in-solution and TCC ligation would be expected to initiate cross-link reversal and liberate cross-linked complexes and small fragments, taking them out of their nuclear context. During ligation in solution, fragment ends would have greater mobility, potentially leading to a fragment length bias. During TCC ligation soluble complexes are immobilized, perhaps leading to a reduced incidence of spurious ligation (and reduced *trans* ratio), but the relaxation of spatial constraints due to nuclear disruption may lead to greater fragment end mobility, which could, for example, lead to a fragment length bias. In contrast, fragment length bias is not observed with in-nucleus ligation (Fig. 5), presumably because fragment end mobility is restricted to a confined nuclear space. This probably explains why we consistently observed more re-ligation for in-nucleus replicates (Table 1; *P* = 0.01, t-test).

## Conclusions

We conclude that in-nucleus Hi-C ligation offers significant improvements over conventional in-solution Hi-C. In-nucleus ligation yields cleaner results with less technical noise characterized by lower ratios of *trans* di-tags. In-nucleus ligation also provides greater reproducibility between replicates at all distance scales, and effectively eliminates fragment length bias. These improvements lead to greater power to discern structural features such as TAD boundaries. Our results suggest that in-nucleus ligation will provide improved results for all experiments employing 3C-based techniques that rely on proximity-dependent ligation (3C, 4C, 5C and Hi-C).

## Materials and methods

### Cells

H9 (WA09; WiCell) human ES cells were maintained using Pluripro fully defined media and matrix (Cell Guidance Systems). Approximately 50 million cells (at passage 56) were harvested with Accutase (Life Technologies), suspended in Pluripro media and directly processed for fixation.

Mouse foetal livers were dissected from C57BL/6 mouse embryos at day 14.5 (E14.5) of development. Foetal liver cells were suspended in DMEM (Dulbecco's modified Eagle minimal essential medium; Life Technologies) supplemented with 10 % foetal bovine serum, filtered through a cell strainer (70 μm) and directly fixed by addition of formaldehyde.

### Hi-C

Except for the ligation step, Hi-C was performed essentially as described in Lieberman-Aiden et al. [15], with some modifications.

Thirty to 50 million cells were fixed in 2 % formaldehyde for 10 min, quenched with 0.125 M glycine, spun down (400 × g, 5 min) and washed once with phosphate-buffered saline. The cells were incubated in 50 ml permeabilization buffer (10 mM Tris–HCl pH 8, 10 mM NaCl, 0.2 % Igepal CA-630, Complete EDTA-free protease inhibitor cocktail [Roche]) for 30 min on ice with occasional agitation, spun down (650 × g, 5 min, 4 °C), and the cell pellets were resuspended in 358 μl of 1.25× NEBuffer2 (NEB) per 5 million cell aliquot. We added 11 μl of 10 % SDS to each aliquot, followed by an incubation at 37 °C for 60 min with continuous agitation (950 rpm). To quench the SDS, 75 μl of 10 % Triton X-100 was then added per aliquot, followed by an incubation at 37 °C for 60 min with continuous agitation (950 rpm). To digest chromatin, 1500 U of HindIII (NEB) was added per aliquot and incubated at 37 °C overnight with continuous agitation (950 rpm). After digestion, restriction sites were filled in with Klenow (NEB) in the presence of biotin-14-dATP (Life Technologies), dCTP, dGTP and dTTP (all 30 μM) for 60 min at 37 °C.

For in-solution ligation, 86 μl of 10 % SDS was added per aliquot and incubated at 65 °C for 30 min with continuous agitation (950 rpm), followed by addition of 7.61 ml of ligation mix (745 μl of 10 % Triton X-100, 820 μl of 10× T4 DNA ligase reaction buffer [NEB], 82 μl of 10 mg/ml bovine serum albumin [NEB] and 5.965 ml water) per aliquot and incubation at 37 °C for 60 min with occasional agitation. For in-nucleus ligation, 7.61 ml of ligation mix (820 μl of 10× T4 DNA ligase reaction buffer [NEB], 82 μl of 10 mg/ml bovine serum albumin [NEB] and 6.71 ml water) was added per aliquot (compared with the in-solution ligation, SDS addition and incubation at 65 °C were omitted). For the ligation reaction (both in-solution and in-nucleus variants), 50 μl of 1 U/μl T4 DNA ligase (Life Technologies) was added per aliquot, followed by incubation at 16 °C for 4 h.

The cross-links were reversed by adding 60 μl of 10 mg/ml proteinase K (Roche) per aliquot and incubating at 65 °C overnight. After overnight incubation, another 60 μl of proteinase K per aliquot was added, followed by incubation at 65 °C for an additional 2 h. RNA was removed by adding 12.5 μl of 10 mg/ml RNase A (Roche) per aliquot and incubating at 37 °C for 60 min. DNA was isolated by a phenol (Sigma) extraction, followed by a phenol/chloroform/isoamylalcohol (Sigma) extraction and standard ethanol precipitation. The precipitated DNA was washed three times with 70 % ethanol, and dissolved in 25 μl TE per aliquot. Subsequently, all aliquots were pooled and the Hi-C DNA was quantified (Quant-iT Pico Green, Life Technologies). Biotin was removed from non-ligated restriction fragment ends by incubating 30–40 μg of Hi-C library DNA with T4 DNA polymerase (NEB) for 4 h at 20 °C in the presence of dATP. After DNA purification (QIAquick PCR purification kit, Qiagen) and sonication (Covaris E220), the sonicated DNA was end-repaired with T4 DNA polymerase, T4 DNA polynucleotide kinase, Klenow (all NEB) and dNTPs in 1× T4 DNA ligase reaction buffer (NEB). Double size selection of DNA was performed using AMPure XP beads (Beckman Coulter), before dATP-addition with Klenow exo^−^ (NEB). Biotin-marked ligation products were isolated with MyOne Streptavidin C1 Dynabeads (Life Technologies) in binding buffer (5 mM Tris pH8, 0.5 mM EDTA, 1 M NaCl) for 30 min at room temperature, followed by two washes in binding buffer, and one wash in 1× T4 DNA ligase reaction buffer (NEB). Paired-end (PE) adapters (Illumina) were ligated onto Hi-C ligation products bound to streptavidin beads for 2 h at room temperature (T4 DNA ligase in 1× T4 DNA ligase reaction buffer [NEB], slowly rotating). After washes in wash buffer (5 mM Tris, 0.5 mM EDTA, 1 M NaCl, 0.05 % Tween-20) and binding buffer, the DNA-bound beads were resuspended in NEBuffer 2. Bead-bound Hi-C DNA was amplified with 12 PCR amplification cycles using PE PCR 1.0 and PE PCR 2.0 primers (Illumina). The concentration and size distribution of Hi-C library DNA after PCR amplification was determined by Bioanalyzer profiles (Agilent Technologies) and quantitative PCR, and the Hi-C libraries were paired-end sequenced on Illumina Hi-Seq 1000 or MiSeq platforms.

### Mapping and filtering

The FASTQ paired-end read data were mapped against the appropriate reference genome (hg19, mm9 or an hg19/mm9 combined genome) and then filtered to remove frequently encountered experimental artefacts using the HiCUP [16] analysis pipeline developed at the Babraham Institute. After the filtering step, we calculated the difference of the ratio of the number of invalid di-tags relative to the uniquely mapped di-tags between the in-nucleus ligation and in-solution ligation datasets. For each di-tag category, we performed a t-test with the null hypothesis that the mean of the differences is 0, that is, there is no difference arising from the ligation step.

### Proportion of hybrid mouse-human di-tags in the hybrid samples

For the mouse-human hybrid samples, we calculated the expected proportion of hybrid mouse-human di-tags (*p*_*hybrid*_) in the Hi-C library, assuming random ligation and that the enzymatic restriction was complete:

$$ {p}_{hybrid}=\frac{2{n}_{fend}^{mouse}{n}_{fend}^{human}}{{\left({n}_{fend}^{mouse} + {n}_{fend}^{human}\right)}^2} $$

where *n*_*fend*_^*mouse*^ is the number of mouse fragment ends (the number of mouse cells multiplied by twice the number of HindIII fragments in the mouse genome, 823,379), and *n*_*fend*_^*human*^ is the number of human fragment ends (the number of human cells multiplied by twice the number of HindIII fragments in the human genome, 837,163). In a sample containing a 5:1 ratio of mouse:human cells, *p*_*hybrid*_ = 0.281.

### Powerlaw curves

We plotted the frequency of *cis*-chromosomal interactions at various genomic distances. The frequency density was obtained by binning the unique *cis*-chromosomal Hi-C di-tags, using 50 bins of equal size on a log_10_ genomic distance plot.

### Bias calculation

We quantified the extent to which the fragment length and the GC content of the fragment ends affect the read coverage using the hicpipe software (version 0.93) [26] developed by Yaffe and Tanay [21]. For each HindIII restriction fragment end, we calculated the fragment length, the GC content of the last 200 bp of the fragment end, and the mappability of the fragment. For the di-tags we used a segment length threshold of 500 bp, that is, we filtered out any di-tags where the sum of the distances from the read positions to the fragment ends where the ligation occurred was greater than this threshold. The algorithm binned the fragment lengths into 20 equally sized bins according to increasing fragment length. In turn, a 20 × 20 interaction matrix of these fragment length bins was used to describe the interaction bias between any two fragment ends. Similarly, a 20 × 20 interaction matrix was constructed using the GC content of the fragment ends. By performing a maximum likelihood optimization using the *trans*-chromosomal data (at 100 kb, 500 kb, 1 Mb and 10 Mb bin resolutions), we obtained the 20 × 20 interaction bias matrices describing the fragment length bias and the GC content bias.

### Normalization of matrices

We calculated the coverage-corrected Hi-C matrices and the coverage-and-distance-corrected Hi-C matrices using the HOMER software [27] employing the algorithm described by Imakaev et al. [22]. It was assumed that the coverage of each bin should be the same in bias-free data, and that the observed Hi-C counts were the true counts multiplied by a factorizable bias (the factorizable bias of two interacting bins was the product of the bias contribution of the two individual bins).

The bias contribution vector and the true interaction matrix were optimized using an iterative approach, starting with the mapped filtered Hi-C data from HiCUP [16]. We used 1 and 10 Mb bin resolutions, excluding bins with coverage less than 20 % of the mean bin coverage, or more than 4 standard deviations away from the mean bin coverage.

### Identification of compartments

We identified the compartments by calculating the first (or, for human samples, the first two) eigenvector(s) of the bin interaction profile correlation matrix for each chromosome, using the HOMER software [27]. The first eigenvector (or, for the human samples, the eigenvector related to the compartmental pattern as opposed to the chromosome arms) was aligned to active histone modification marks. This was done by multiplying the eigenvector by −1 if the Pearson correlation coefficient of the eigenvector and the H3K4me3 histone modification mark ChIP-seq [19, 28] profile was negative. The magnitude of the correlation coefficient was typically around 0.7. Chromosome bins with positive values in the eigenvector were considered to be in the A compartment, and bins with negative values to be in the B compartment. For the human chromosome 4, there was no clear separation between the first and second eigenvector profiles, so reads on human chromosome 4 were omitted from further analyses.

### Compartment interaction bias among mouse–human hybrid reads

For the hybrid mouse–human di-tags, we assessed if there were any compartment-dependent non-random interactions, for instance, if mouse compartment A formed interactions preferentially with human compartment A. We counted hybrid di-tags in which both reads mapped to either compartment A or compartment B. We performed Fisher’s exact test on these counts.

### Scatter plots and measures of matrix reproducibility

We calculated the Spearman correlation of all *cis*- and *trans*-chromosomal interactions between different Hi-C experiments, at a 10-Mb bin resolution, as well as at a TAD level, using TADs as variable sized bins. In addition, we plotted each binned interaction count in one dataset against the corresponding interaction count in a second dataset. We colored the points of the plot according to the genomic distance of the interacting bins.

We subdivided the bin interaction count data according to the genomic distance of the interacting bins, and performed a linear fit on each of these datasets (y = ax + b, where a is the slope and b is the intercept). For each distance, we then corrected the slope for the Hi-C library sizes (a_corr_ = a C_x_/C_y_ where C_x_ and C_y_ are the total counts in the libraries shown on the x and y axes). The DES was then the angle between the corrected slope and the y = x line:

$$ \mathrm{D}\mathrm{E}\mathrm{S} = \mathrm{atan}\left({\mathrm{a}}_{\mathrm{corr}}\right)\ \hbox{-}\ \mathrm{atan}(1). $$

A perfectly reproducible experiment would result in DES = 0 and a Spearman correlation R = 1.

### Calculation of TAD boundaries

We calculated TADs in our coverage-corrected Hi-C matrices using the Hi-C domain finding tool of the HOMER software [27]. The algorithm defined directionality indices (DIs) as described in [18], based on the ratio of upstream and downstream interaction counts. We quantified the number of upstream and downstream interactions within an interaction distance of 1 Mb, using 25-kb overlapping bins with a step size of 5 kb. Bins with coverage less than 15 % of the mean bin coverage or greater than 4 standard deviations above the mean were excluded. This resulted in DI values at an effective 5-kb resolution (at the centre of each 25-kb window), which were further smoothed using a running average over a ±25 kb window. Domain boundaries were then called where the smoothed DI was at a local extremum and at least 0.5 standard deviations away from the mean. Using the domains identified by HOMER, we called consensus TAD boundaries for in-solution ligation and in-nucleus ligation datasets, by keeping only TAD boundaries (rounded to the closest genomic position using a 25-kb resolution).

### Hi-C interactions around TAD boundaries

We plotted the interaction directionality profile around the TAD boundaries using the average of the standard scores of the un-smoothed DI values, as a function of distance from the domain boundary upstream or downstream. A random control included 9686 randomly selected genomic positions. In addition, we plotted the coverage- and distance-corrected Hi-C interaction profiles around the consensus TAD boundaries using HOMER [27] and 25-kb overlapping bins with a step size of 5 kb.

### Availability of supporting data

The datasets supporting the results of this article are available in the Gene Expression Omnibus (GEO) repository under accession number [GEO:GSE70181] [29].

## Additional files

Additional file 1:
**Schematics of in-solution ligation and in-nucleus ligation Hi-C experimental protocols.** (PDF 746 kb) Additional file 2:
**Assessing randomness of hybrid di-tags.** (PDF 52 kb) Additional file 3:
**In-nucleus versus in-solution ligations.** (PDF 337 kb)

## Abbreviations

3C: chromosome conformation capture
4C: circularized chromosome conformation capture
5C: carbon-copy chromosome conformation capture
bp: base pair
DES: deviation from the expected slope
DI: directionality index
ES: embryonic stem
INL: in-nucleus ligation
ISL: in-solution ligation
kb: kilobase
Mb: megabase
PCR: polymerase chain reaction
PE: paired-end
SDS: sodium dodecyl sulfate
TAD: topologically associated domain
TCC: tethered conformation capture
