# Supplementary material for: Comparison of Hi-C results using in-solution versus in-nucleus ligation
Source: Genome Biol. 2015 Aug 26;16(1):175. doi: 10.1186/s13059-015-0753-7 (PMC4580221; doi:10.1186/s13059-015-0753-7)
Supplement: Additional file 1: — Schematics of in-solution ligation and in-nucleus ligation Hi-C experimental protocols. (PDF 746 kb) [file 13059_2015_753_MOESM1_ESM.pdf]

Additional File 1. Schematics of in-solution ligation and in-nucleus ligation Hi-C experimental protocols

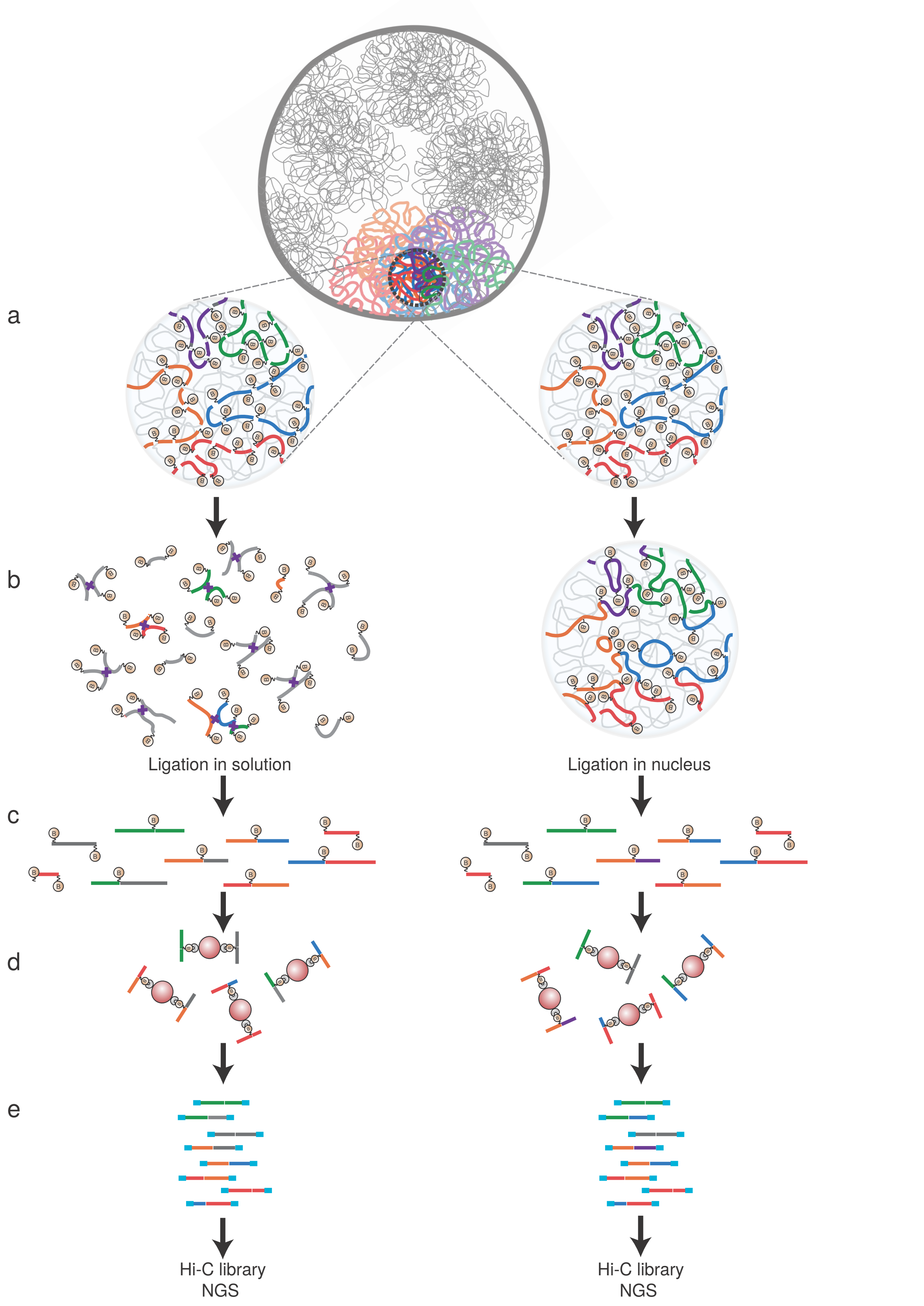

a) Enlarged region of cross-linked nuclei digested with restriction enzyme and filled in with biotin (B, yellow balls).Coloured curves represent chromatin/DNA from a chromosome territory. **b)** Ligation in solution versus in nucleus. **c)** Biotinylated ligation junctions after ligation and sonication. **d)** Capture of biotinylated ligation junctions on streptavidin-coated magnetic beads. **e)** PCR amplified products ready for sequencing.
