# Supplementary material for: Comparison of Hi-C results using in-solution versus in-nucleus ligation
Source: Genome Biol. 2015 Aug 26;16(1):175. doi: 10.1186/s13059-015-0753-7 (PMC4580221; doi:10.1186/s13059-015-0753-7)

Additional File 2. Assessing randomness of hybrid di-tags.

We calculated if there is non-randomness between A and B compartment interactions among the hybrid di-tags, by performing a Fisher test on the di-tag counts, using a 1Mb bin resolution for the compartments.

sample	unique di-tags	A_m-A_h	A_m-B_h	B_m-A_h	B_m-B_h	P_{Fisher}
mouse-human-1 ISL	17,137,291	408,084	549,043	393,004	525,479	0.035
mouse-human-2 ISL	488,340	2,851	3,976	2,572	3,618	0.82
mouse-human-1 INL	11,533,994	1,610	2,158	1,641	2,260	0.56
mouse-human-2 INL	573,366	82	82	77	78	1.00

The mouse-human-1 INL, mouse-human-2 INL and mouse-human-2 ISL show no statistically significant bias for compartmental interactions. Although the mouse-human-1 ISL dataset shows statistically significant non-random A-B compartment interactions, this is only a statistical significance due to the large number of counts, and does not imply biological significance (the odds ratio is 0.994, with a 95% confidence interval of (0.988, 0.9996)). Error bars show 95% confidence interval.

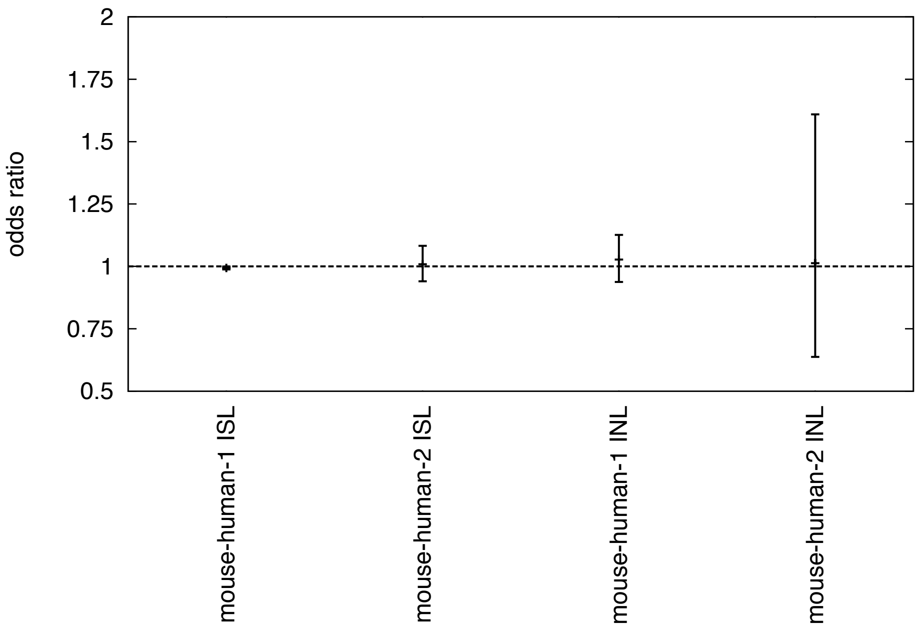

Supplement: Additional file 2: — Assessing randomness of hybrid di-tags. (PDF 52 kb) [file 13059_2015_753_MOESM2_ESM.pdf]
