# Supplementary material for: Comparison of Hi-C results using in-solution versus in-nucleus ligation
Source: Genome Biol. 2015 Aug 26;16(1):175. doi: 10.1186/s13059-015-0753-7 (PMC4580221; doi:10.1186/s13059-015-0753-7)
Supplement: Additional file 3: — In-nucleus versus in-solution ligations. (PDF 337 kb) [file 13059_2015_753_MOESM3_ESM.pdf]

Additional File 3. In-nucleus versus in-solution ligations.

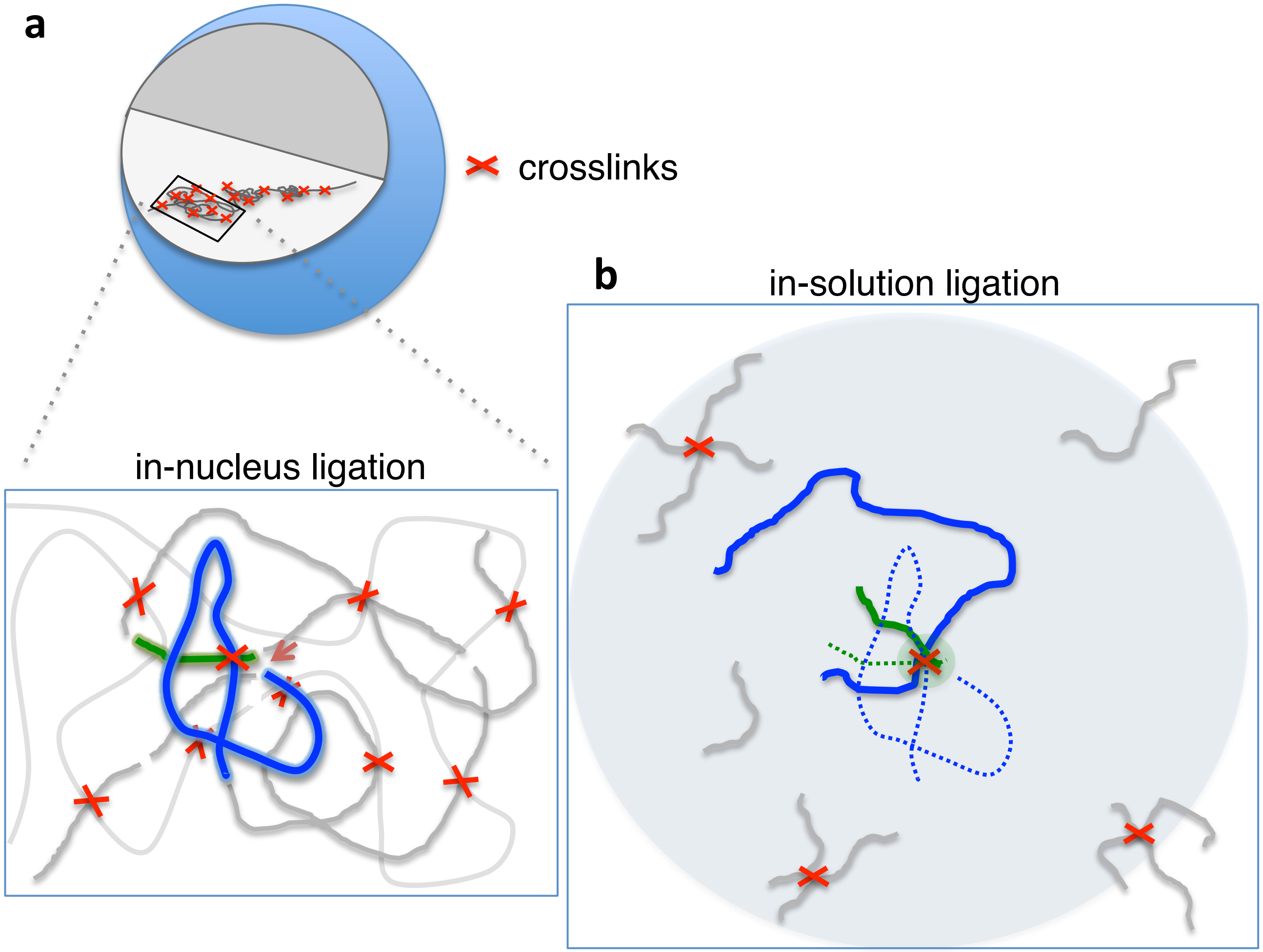

In-nucleus versus in-solution ligations. a) During in-nucleus ligation, chromatin fragments are fixed in position and ligate to other fragment ends in close nuclear proximity. **b)** With in-solution ligation nuclear structure is lost and fragment end mobility is increased. Solid green and blue lines represent possible conformations of the original cross-linked complex (dashed lines). Light blue and green circles depict the potential volumes explored by the blue and green fragment ends shown by the orange arrow in **a**). This difference in mobility may explain fragment length bias observed with in-solution ligation. In-solution ligation takes place in greater than one million fold excess of complexes and fragments from other chromosomes and/or other cells (grey lines) leading to increased spurious ligation products manifest as increased trans di-tags and experimental noise.
